# Truthful but Misleading: Advanced Linguistic Strategies for Lying Among Children

**DOI:** 10.3389/fpsyg.2020.00676

**Published:** 2020-04-24

**Authors:** Chao Hu, Jinhao Huang, Qiandong Wang, Ethan Weare, Genyue Fu

**Affiliations:** ^1^Institute of Psychological Sciences, Hangzhou Normal University, Hangzhou, China; ^2^Art Therapy Psychological Research Centre, Hangzhou Normal University, Hangzhou, China; ^3^Zhejiang Key Laboratory for Research in Assessment of Cognitive Impairments, Hangzhou Normal University, Hangzhou, China; ^4^Peking-Tsinghua Center for Life Sciences, Peking University, Beijing, China; ^5^Department of History, University of Toronto, Toronto, ON, Canada

**Keywords:** lying, linguistic strategy, development, child education, grounded theory

## Abstract

We explored whether children could apply linguistic strategies for lying, i.e., manipulating linguistic content of speech to mislead others. We announced a knowledge-test entailing prizes in the classrooms of a primary school and a middle school. Altogether 79 Chinese children (6–18 years) voluntarily participated in the test: listening to a series of animal sounds before guessing the names of the animals. Meanwhile, behind the participants, a video was playing images that ostensibly corresponded to the sounds being played. In fact, this was not necessarily the case, i.e., some items cannot be solved because the sounds played are not from any animal but machine-synthesized. Participants were instructed not to look back at the video. However, 51 children peeked at the video for the unsolvable items, although the peeking behavior decreased with age. Moreover, when explaining how they correctly guessed the unsolvable items, children as young as 6 years old were able to apply a linguistic strategy (i.e., “capability attribution”) for lying. Besides “capability attribution,” Children also applied “fortune attribution” and “topic shift” for lying. Finally, “fortune attribution” and “topic shift” increased with age. Therefore, educators need to be aware that children are able to apply verbal strategies for lying that could involve truthful statements (i.e., “topic shift”) or statements that are difficult to be proved as untruthful (i.e., “fortune attribution”).

“*Truthful is not equal to Honest*.”—*Yi Zhongtian, Why I trust Zhang Wenhong (A Doctor fighting the COVID-19)*.

The existence of lies increases the uncertainty and complexity of human language. DePaulo et al. (2003) defined lying as the deliberate misleading of others. Lying could significantly harm common welfare (Prooijen and van Lange, 2016). However, children begin to lie as early as 2 years old (Lee, 2013; Williams et al., 2017). A laboratory study showed that children between 6 and 11 years old were not good at maintaining consistency between initial lies and subsequent verbal statements (Talwar et al., 2007). However, an ecological study showed that children’s everyday deceptions reported by mothers were varied, flexible, context-appropriate, and complex, demonstrating deception skills developed from pragmatic need and situational exigencies (Newton et al., 2000).

In real life, most lying is not an effortful fabrication of untruthful statements, but effortless and covert manipulation of language as a contextual problem-solving activity driven by the desire for quick, efficient, and viable communicative solutions for their reputations and interests (McCornack et al., 2014). Linguistic strategies are the ways speakers manipulate their linguistic content of speech for their purpose. Consistently, linguistic strategies for lying are the ways liars manipulate the linguistic content of speech to mislead others, which could involve truthful statements. For example, if one asks a salesman whether ginseng is good for one’s health, the salesman could say: “People who eat ginseng every day live longer than others.” This may be true, but it is irrelevant to the question (i.e., “topic shift”) and is misleading. (People who eat expensive ginseng every day are wealthy and thus live better). A study demonstrated that 5-year-old children judged false implicatures (e.g., telling a truth: “she is smiling at you.” when the speaker knows that she is actually angry at you) as lies (Antomo et al., 2018).

More advanced linguistic strategies could involve truthful statements or statements that are difficult to be proved as untruthful. For example, if a teacher suddenly asked a student who cheated on an exam, “What did you do to make you excel in this exam?” The student could reply, “I worked very hard,” (i.e., “capability attribution”) or “I was just lucky” (“fortune attribution”). The strategy of “fortune attribution” is more advanced because the teacher could never find out if the student were not fortunate in the exam, but the teacher could find out if the student were hard-working.

Years of lying, lie-detection, and anti-lie-detection practices may promote the development of linguistic strategies among children. Investigating the development of linguistic strategy among children could help develop honesty-endorsement education programs in the school. Moreover, Meibauer (2018) believed that applied linguistics should profit from a lying study producing semantic data and more fine-grained evaluations of lying, e.g., therapy for pragmatic impairment to language and the law to the study of persuasion and propaganda. Finally, pragmatic lie-detection may benefit from studying the development of linguistic strategies for lying.

However, few studies have investigated children’s linguistic strategies for lying. Most studies on lying focused on simple forms of lies, e.g., pointing at an empty box/wrong card to mislead adults who tried to get chocolates/right cards (e.g., Russell et al., 1994; Smith and LaFreniere, 2013; Zhao et al., 2017; Sai et al., 2018a, b), concealing the truth (e.g., Talwar and Lee, 2008; Evans and Lee, 2011), or reporting fake dice points (e.g., Gächter and Schulz, 2016).

The traditional temptation-resistance paradigm has been widely used in the research of lying among children for its advantages in bringing about spontaneous lying and ecological validity. Lewis et al. (1989) used a temptation resistance paradigm in which they told the three-year-olds not to peek at a toy when the experimenter left the room. The majority of children peeked due to the highly tempting nature of the situation. As soon as children peeked at the toy, the experimenter returned to the room and asked the children if they had peeked. Among the three-year-olds who peeked, 38% lied by denying they had peeked, 38% confessed, and 24% gave no verbal response. In another study, half of the three-year-olds confessed to their transgression, whereas most seven-year-olds lied (Talwar and Lee, 2002).

Further research on children between 6 and 11 years of age in a similar paradigm (i.e., a trivia knowledge-test: children peeking at the answer to a trivia question while left alone in a room) showed that transgression decreased with age; moreover, lying did not increase with age (Talwar et al., 2007). Another study found that older children were able to devise highly plausible explanations for their knowledge (Talwar and Lee, 2008). Finally, Evans and Lee (2011) found that lying was less prevalent among 41 eleven- to thirteen-year-old and 34 fourteen- to sixteen- year old children than 33 eight- to ten-year-old children; however, no significant age difference was found for the “sophistication of lies” (i.e., “ability to maintain their lie by concealing incriminating knowledge they ought not to know”). Note that all these studies were conducted with Western children.

Despite the accumulated evidence about lying among Children, most studies focused on children’s concealing of the truth (which is an untruthful statement), without investigating linguistic strategies for lying (which could involve truthful statements). Moreover, there is no study on linguistic strategies for lying among Chinese children. To investigate the linguistic strategies for lying among Chinese Children aged 6–18, we applied an adapted temptation resistance paradigm: some items in the knowledge-test are impossible to be solved without transgression, and the transgressed children were asked how they responded correctly on these unsolvable items. Although the temptation resistance paradigm used in this study also involved eliciting a form of “truth-concealing” from participants, our focus was on the question about how they solved the unsolvable items, which might elicit truthful but misleading statements.

This study may inspire researchers studying Chinese language learning, few of whom have paid attention to children under 18 (for reviews, see Ma et al., 2017; Gong et al., 2018, 2020). Linguistic strategies for lying may develop with age due to the development of language use. In addition, due to gender differences in children’s language use (for a review, see Leaper and Smith, 2004), there may be some gender differences in the linguistic strategy for lying.

## Research Question

Can Chinese children apply a linguistic strategy for lying? Which strategies do they use?

## Methods

The ethics committee at the sponsoring university approved all procedures used in the current study. Informed consent from parents of the children was obtained via communication between the parents and the teachers before the conduction of this study. Moreover, participants were recruited with the informed consent of the participants’ headmasters and teachers. Finally, informed consent was obtained from participants after thoroughly explaining the nature and consequence of this study at the beginning of the test.

### Participants

The experimenter went to a primary school and a middle school in Jinhua, a city of 3 million. The experimenter announced the knowledge-test orally in the participants’ classrooms during the break of the classes. Students then raised their hands to express interest in the test. Each time, the experimenter randomly selected a child to test. The selected child followed the experimenter to another quiet classroom for testing.

Altogether 79 native-born Chinese students (38 females, 41 males; 6–18 years old, *M* = 12.78 years, *SD* = 3.92 years) participated voluntarily. All children were typically developing and did not report any visual impairment. Each participant was compensated with a prize of an item of stationery costing around 10 RMB (about 1.5 US dollars) for their participation.

### Material

***A Lenovo Think-pad laptop*,** with a built-in camera (30 fps). The indicator light of the camera was hidden by a cartoon sticker, and thus the participants were not able to notice the operation of the camera throughout the experiment.

***A video of 13 different animals***: Dog, cat, and cow in the earlier part serving for the practice trials; another 10 animals in the later part (goat, duck, dolphin, rooster, mouse, woodpecker, leopard, snake, sika deer, and dinosaur) served for the experimental trials. In each experimental trial, an animal picture was displayed for 15 s, accompanied by its sound. However, the accompanying sound did not necessarily come from the animal. In particular, the sounds for dolphin, woodpecker, leopard, sika deer, and dinosaur were unrelated to any animal (unsolvable items). We did this in order to lure the participants into peeking at the pictures displayed in the video. To control the confounding of knowledge about animals, we selected the solvable items according to the education of the youngest children; besides, the sounds of unresolvable items are synthesized by machines and do not belong to any animal.

### Procedure

The participants finished the experiment individually in quiet classrooms on their campus. After the participants sat down at a desk, they were instructed to finish a “listen and guess the animal” test: the more animals they guessed correctly, the better the prize they would receive. Considering the potential effect of expectation on motivation, participants did not know what the prizes were until they finish the experiment. The laptop showing the video of the animals was placed on the desk behind the participants. Participants listened to the sounds from the video and guessed the animals’ names, writing down their answers on a paper.

Before the experimental trials, the participants practised guessing dog, cat, and cow (the sounds of which were familiar to the participants). After the participants correctly guessed all the animals’ names, the experimenter asked them to turn their heads around to look at the pictures in the video. We made this arrangement to ensure that the participants believed the corresponding animals were displayed on the laptop. All the participants correctly guessed the animals in the practice trials and then proceeded to the experimental trials.

Before the experimental trials, the experimenter instructed them not to peek at the video. When the picture of “Goat” was displayed in the experimental trials, the experimenter’s cellphone rang (according to the intended plan for the experiment). Then the experimenter walked out of the classroom, explaining that he had to step out for a phone call, and instructed the participants not to look at the video displaying the animal. About 2–3 min later, the experimenter returned to the classroom, by which time the experimental trials were finished.

The experimenter went ahead and sat on the chair behind the laptop, asked the participants to turn around and look at him, so that the participants were facing the built-in camera which was secretly videotaping the participants’ actions throughout the experiment.

The experimenter asked: “While I was out, did you peek at the screen?”

After the participants responded to this question about peeking, the experimenter asked them to hand over the paper and then checked the participant’s answers. For each of the unsolvable items (i.e., dolphin, woodpecker, leopard, sika deer, and dinosaur) a participant had written, the experimenter commented it was difficult to guess the animal, and asked, “How did you guess correctly?” The experimenter asked this question more than once if a participant had answered correctly on more than one unsolvable item.

After the experiment, the participant received a randomly chosen gift regardless of her/his performance in the experimental trials.

### Data Analyses

Frequency of cheating (i.e., times of turning around one’s head to peek) and lying (i.e., denial of turning around one’s head to peek) were coded for each participant by two independent coders through watching the videos recorded by the camera. See the Supplementary Material for the original data.

We used Grounded Theory to identify common linguistic strategies the participants used when accounting for their correct answers to the unsolvable items, applying the same approach used in the same context before (Hu et al., 2018). Below are the rationales for applying Grounded Theory: verbal strategies for lying are dynamic structures of the language used by speakers during their ongoing interaction with their audiences, while the Grounded theory is for phenomena that “are not conceived of as static but as continually changing in response to prevailing conditions” (Corbin and Strauss, 1990). Moreover, unlike most studies on lying in which responses are categorized dichotomously into truth-telling or lying, verbal strategies for lying could take many different forms and involve truthful statements, the speakers make “choices according to perceived options” (Corbin and Strauss, 1990). Finally, children in this study were seen as “having, though not always utilizing, the means of controlling their destinies by their responses to conditions” (Corbin and Strauss, 1990); therefore, this study did not only aim to “uncover relevant conditions but also to determine how the actors under investigation actively respond to those conditions” (Corbin and Strauss, 1990).

Below are the steps of our method: first, two graduate students majoring in psychology read the whole procedure of this study. Second, they read the original transcript of all the participants’ responses. Third, they independently labeled each response with a provisional conceptual term (e.g., “learned by watching TV”) in Excel 2010. If a response is perceived as resembling the same conceptual term proposed before, then the response is labeled with that conceptual term; otherwise, a new conceptual term should be proposed. A response could be labeled with multiple terms, listed on multiple columns in Excel. Fourth, after labeling all the responses, the graduate students used the “filter” in Excel to compare all the responses under the same conceptual term, and then across different conceptual terms. A term would be discarded unless it stood up to continued scrutiny through its repeated proven relevance to the phenomenon under question. Conceptual terms (e.g., “learned by watching TV,” “learned by reading books”) pertaining to the same phenomenon were grouped to form the same categories (e.g., “capability attribution”), which are higher-level, more abstract concepts. Fifth, the first author of this report read the concepts and all the responses under each concept and then discussed with the graduate students for new insights and increased theoretical sensitivity. Conceptual terms were revised (e.g., “lucky” to “fortune attribution”) to systematically resemble the full range of variation of the phenomena. A codebook was then developed based on the final conceptual terms. Finally, two trained coders coded each participant’s linguistic strategy for lying according to the codebook. For all the codes, coder agreements were higher than 80%, kappa values higher than 0.70. The final frequency of each code was obtained after the coders reached consensus through a discussion about the initial disagreements.

## Results

### Cheating and Lying

Altogether, 51 participants (of the 79 participants) had cheated, turning their heads around to peek, and 42 participants (of the 51 transgressed participants) lied when responding to the question about cheating. Moreover, children as young as 6 years old were able to apply a linguistic strategy for lying, making up plausible reasons (e.g., “I have watched it on Animal World”) to explain the impossible success at guessing the unsolvable items.

Linear regression analysis with age and gender as the predictors was conducted on the frequency of cheating for all the participants. The model was significant, *F*(2, 76) = 16.97, *p*< 0.001, Adjusted *R*^2^ = 0.29. Age effect was significant, *B* = −0.47, *t* = −5.60, *p*< 0.001, *95%* CI for *B* = [−0.64, −0.30]. Frequency of cheating decreased with age. Gender effect was not significant, *p* > 0.05.

Logistic regression analysis choosing age and gender as the predictors was conducted on the dichotomous variable lying (0–confession, 1 - lying) among the participants who cheated. The model was marginally significant, χ^2^ = 5.07, *df* = 2, *p* = 0.079. Age was a significant predictor, *B* = −0.22, *Wald* = 4.35, *df* = 1, *p* = 0.037, *95%* CI for *Exp(B)*: (0.65, 0.99). Lying decreased with age. Gender effect was not significant, *p* > 0.05.

### Linguistic Strategies for Lying When Justifying Correct Answers to Unsolvable Items

Three linguistic strategies for lying used by the transgressed participants were identified: (1). “capability attribution”: rationalizing their correct answers through explaining their cognitive capability, e.g., “watching Animal World (a TV program),” or explaining their reasoning process, e.g., “It is neither a tiger nor a lion. Thus I thought it was likely a leopard. I inferred it by comparing it with other animals.” (2) “fortune attribution”: attributing a correct answer to a lucky guess. For example, “I just guessed it.” (3) “topic shift”: mentioning something irrelevant to the question, e.g., “The sound was not clear.” Note that five participants confessed when asked if they had peeked, but applied verbal strategies for lying to justify correct answers to unsolvable items. See Table 1 for the numbers of participants applied different verbal strategies for lying.

**TABLE 1:** Linguistic strategies for lying when justifying correct answers to the unsolvable items among the Children confessed/concealed transgression.

Response to the 1st question	Capability attribution	Fortune attribution	Topic shift
Confessed peeking (truth-told)	3	0	2
Concealed peeking (lied)	34	13	6
Total	37	13	8

Logistic regression analysis choosing age and gender as the predictors was conducted on each dichotomous variable of linguistic strategy (0- absent, 1- present). The results showed that the model was significant on “fortune attribution”, χ*^2^* = 8.16, *df* = 2, *p* = 0.017, *Nagelkerke R*^2^ = 0.24. Age was a significant predictor, *B* = 0.25, *Wald* = 6.12, *df* = 1, *p* = 0.013, *95% Confidence Interval for Exp(B)*: [1.05, 1.56]. The model was also significant on “topic shift”, χ*^2^* = 6.30, *df* = 2, *p* = 0.043, *Nagelkerke R*^2^ = 0.22. Age was a significant predictor, *B* = 0.26, *Wald* = 4.85, *df* = 1, *p* = 0.028, *95% Confidence Interval for Exp(B)*: [1.03, 1.63].

Gender was not a significant predictor for any linguistic strategy, all *p* > 0.05. See Table 2 for the detailed results of the logistic regression analyses.

**TABLE 2:** Results of the logistic regression analyses choosing age and gender as the predictors on each linguistic strategy.

Dependent variable	Predictors	B	S.E.	Wald	df	Sig.	Exp(B)	95% C.I. for EXP(B)	
								Lower	Upper
Capability attribution	Age	0.031	0.112	0.080	1	0.778	1.032	0.829	1.284
	Gender	20.675	N.A.	0.000	1	0.998	N.A.	0.000	
	Constant	0.190	1.376	0.019	1	0.890	1.209		
Fortune attribution	Age	0.248	0.100	6.123	1	0.013	1.282	1.053	1.561
	Gender	–0.522	0.736	0.504	1	0.478	0.593	0.140	2.509
	Constant	–3.621	1.375	6.940	1	0.008	0.027		
Topic shift	Age	0.259	0.118	4.845	1	0.028	1.295	1.029	1.631
	Gender	–0.415	0.867	0.229	1	0.632	0.660	0.121	3.613
	Constant	–4.560	1.723	7.003	1	0.008	0.010		

## Discussion

Overall, our results demonstrated three linguistic strategies for lying applied by children to account for their impossible success in the knowledge-test, i.e., “capability attribution,” “fortune attribution,” and “topic shift.” Moreover, the older children are more likely to conceal their transgression by “fortune attribution” and “topic shift” than the younger children. Nevertheless, “capability attribution” was the most commonly identified linguistic strategy among our participants. Consistently, Talwar and Lee (2008) found that older children were able to devise highly plausible explanations for their knowledge. Our results further suggested that “capability attribution” was not the only strategy children applied for lying. More advanced linguistic strategies for lying, such as “fortune attribution” and “topic shift,” continued to develop with age. The development in the linguistic strategy for lying is probably due to the years of practice in lying, lie-detection, and anti-lie-detection.

Consistent with the study among Canadians by Evans and Lee (2011), transgression and lying among Chinese children declined with age. However, our study showed that all of the participants who cheated applied some linguistic strategy when responding to the experimenter’s further questioning. Note that this study investigated children’s answers to open-ended questions rather than binary answers to truth/lie questions, adopting a Grounded theory method to investigate children’s choices according to their “perceived options” (Corbin and Strauss, 1990). Children in this study “are seen as having, though not always utilizing, the means of controlling their destinies by their responses to conditions” (Corbin and Strauss, 1990); therefore, this study demonstrated how children “actively respond to those conditions” (Corbin and Strauss, 1990). As a result, creativity in children’s verbal strategies was exposed rather than hidden. Similarly, a previous study applying an open question design demonstrated the development of non-verbal tactical deception among children (Smith and LaFreniere, 2013).

Six participants who had confessed transgression lied about the degree of their transgression (i.e., the number of times they had peeked) with immediate follow-up explanations. Moreover, five children confessed transgression but lied about how they got the answers to the unsolvable items. Therefore, participants cannot be categorized dichotomously into liars and truth-tellers. These results supported the Information Manipulation Theory proposed by McCornack et al. (2014): even after a confession, an individual could still manipulate partial information covertly along multiple dimensions and as a contextual problem-solving activity driven by the desire for quick, efficient, and viable communicative solutions for their reputations and interests.

### Limitation

The cross-section nature of this study did not allow a developmental trajectory to be drawn from the individual child’s use of linguistic strategies for lying. Ideally, a longitudinal design would serve such purpose adequately.

## Conclusion

Children as young as 6 years old were able to apply a linguistic strategy for lying. Chinese children applied three linguistic strategies for lying when justifying correct answers to unsolvable items, i.e., “capability attribution,” “fortune attribution,” and “topic shift.” Besides, cheating and lying decreased, whereas strategies of “fortune attribution” and “topic shift” increased from 6 to 18 with age. Children’s linguistic strategy for lying may develop through years of lying, lie-detection, and anti-lie-detection practices; after learning that telling untruthful statements is risky. Therefore, educators need to be aware that children are able to apply verbal strategies for lying that could involve truthful statements (i.e., “topic shift”) or statements that are difficult to be proved as untruthful (i.e., “fortune attribution”).

## Data Availability Statement

All datasets generated for this study are included in the article/Supplementary Material.

## Ethics Statement

The studies involving human participants were reviewed and approved by the Ethics Committee at Zhejiang Normal University. Informed consent to participate in this study was provided by the participants’ legal guardian/next of kin.

## Author contributions

CH developed the idea and designed the study. QW collected the data. JH analyzed the data. CH and JH wrote the main manuscript text. EW helped with the writing. GF contributed to the concept. All authors reviewed the manuscript.

## Conflict of Interest

The authors declare that the research was conducted in the absence of any commercial or financial relationships that could be construed as a potential conflict of interest.

## Supplementary Material

DATA SHEET S1 Data of children’s strategy for lying.
